# The Brain Alteration of Seafarer Revealed by Activated Functional Connectivity Mode in fMRI Data Analysis

**DOI:** 10.3389/fnhum.2021.656638

**Published:** 2021-04-22

**Authors:** Yuhu Shi, Weiming Zeng, Nizhuan Wang

**Affiliations:** ^1^College of Information Engineering, Shanghai Maritime University, Shanghai, China; ^2^Artificial Intelligence and Neuro-Informatics Engineering (ARINE) Laboratory, School of Computer Engineering, Jiangsu Ocean University, Lianyungang, China

**Keywords:** functional magnetic resonance imaging, activated brain voxel, functional connectivity, support vector machine, seafarer

## Abstract

As a special occupational group, the working and living environments faced by seafarers are greatly different from those of land. It is easy to affect the psychological and physiological activities of seafarers, which inevitably lead to changes in the brain functional activities of seafarers. Therefore, it is of great significance to study the neural activity rules of seafarers’ brain. In view of this, this paper studied the seafarers’ brain alteration at the activated voxel level based on functional magnetic resonance imaging technology by comparing the differences in functional connectivities (FCs) between seafarers and non-seafarers. Firstly, the activated voxels of each group were obtained by independence component analysis, and then the distribution of these voxels in the brain and the common activated voxels between the two groups were statistically analyzed. Next, the FCs between the common activated voxels of the two groups were calculated and obtained the FCs that had significant differences between them through two-sample *T*-test. Finally, all FCs and FCs with significant differences (DFCs) between the common activated voxels were used as the features for the support vector machine to classify seafarers and non-seafarers. The results showed that DFCs between the activated voxels had better recognition ability for seafarers, especially for Precuneus_L and Precuneus_R, which may play an important role in the classification prediction of seafarers and non-seafarers, so that provided a new perspective for studying the specificity of neurological activities of seafarers.

## Introduction

It is a very meaningful research hotspot using functional magnetic resonance imaging (fMRI) to explore the relationship between neural activity of the brain and individual behaviors and further consider how they are influenced by the occupation-related training or experiences in the field of cognitive neuroscience. Compared with the traditional methods for studying brain neural activity, fMRI is an emerging research technology with advantages of non-radiation, non-invasion, as well as higher temporal and spatial resolution (Poldrack et al., 2011), which has been widely adopted by the neuroscience community. This is primarily because it allows researchers to unobtrusively sample patterns of neural activity across the entire human cerebral cortex on a more fine-grained spatial scale, usually just a few millimeters (Finn et al., 2015; Dubois and Adolphs, 2016; De Vos et al., 2018).

In particular, the resting-state fMRI modality provides a new opportunity for the study of the brain’s intrinsic activities. Through resting-state fMRI-based brain functional connectivity (FC) analysis, a detailed and comprehensive description of brain tissue patterns can be achieved, which is helpful to reveal the neural mechanism of cognitive process and the pathogenesis of various neuropsychiatric diseases (Rosenberg et al., 2016; Cui and Gong, 2018; Nielsen et al., 2018). Therefore, it has been attracted more and more attention from brain researchers in recent years, where FC refers to the temporal correlation between spatially separated brain regions, and any way that measures the correlation between two time series can be used to characterize FC (Dijk et al., 2010; Calhoun and Adali, 2016; De Lacy et al., 2018).

Currently, the main research methods of FC include correlation analysis based on seed point, independent component analysis (ICA), sparse component analysis, non-negative matrix factorization, and cluster analysis (Shi et al., 2015a; Cohen et al., 2017; Gong et al., 2018; Varikuti et al., 2018). These methods can be roughly divided into two categories: one is model-driven method, which requires the researchers to select the regions of interest in advance, so the researchers need to have some prior knowledge (Friston et al., 1994; Sun et al., 2004; Smitha et al., 2017). The other is data-driven method, which does not need any prior knowledge, and the whole-brain FC pattern can be obtained, but the interpretation of the results is not as intuitive as the seed point-based correlation analysis (Alexander and Baumgartner, 2001; Frey and Dueck, 2007; Li et al., 2017; Shi et al., 2018).

So far, many occupations have been studied in the fMRI field including musicians, expert athletes, acupuncturists, simultaneous interpreters, taxi drivers, composers, etc. (Schlaug et al., 1995; Park et al., 2009, 2011; Wei et al., 2011; Dong et al., 2015). For example, Dong et al. (2013) found that acupuncturists had a higher amplitude of low-frequency fluctuations in the left ventral medial prefrontal cortex and in the contralateral hand representation of the primary somatosensory area compared to matched non-acupuncturists. Hervais-Adelman et al. (2014) revealed that the brain functional plasticity was related to the emergence of extreme language control expertise by exploring the functional response of participants with simultaneous interpretation training during fMRI scanning. Besides, Shen et al. (2016) showed that there was a significant difference between the professional taxi drivers and non-taxi drivers in the FC patterns of early warning function network, and a distinguish rate of 90% between them was obtained by using these differences.

As a special professional group, seafarers are often engaged in their own work with single-gender colleagues (male) for a long time and exposed to the marine working environment such as narrow working space with a high level of machine noise, as well as long periods of isolation from their families. Additionally, this job requires professional maritime training and skills, strong ability to adapt to the environment and to obey a chain of commands, as well as good psychological quality (Wang et al., 2017; Shi and Zeng, 2018). The occupational stability and particularities make that seafarers have their specific individual behaviors and career experiences, which lead to specific changes in the brain neural activity. These changes not only influence the physical and psychological health of seafarers themselves but also seriously affect the safety of navigation operation (Shi et al., 2019). Therefore, it is very important to deeply study the neural activity changes of the seafarer’s brain.

Recently, Shi et al. (2015) proposed a kind of seafarers’ psychological health assessment method based on the technology of fMRI. Support vector machine (SVM) is used as the classifier to implement the binary classification of seafarers’ fMRI data without labels through a detailed study of default mode network (DMN). The significant difference between these two types of seafarers is verified, which causes the extensive concern of the shipping industry (Shi et al., 2015b). Wang et al. (2018) explored the functional complexity changes of seafarers’ brain based on fMRI and a brain entropy model, where the sample entropy was used to reveal the brain’s complexity. The results showed that the entropy of orbital-frontal gyrus and superior temporal gyrus in the seafarers was significantly higher than that in the non-seafarers. The entropy of the cerebellum in the seafarers was lower than that of the non-seafarers, which implied that the seafarer occupation indeed impacted the brain’s complexity (Wang et al., 2018).

However, most of these studies do not take full advantage of the high spatial resolution of fMRI data and only consider the aggregation characteristics of voxel groups. For example, the simple statistical relationships (such as correlations) between the average time series of all voxels in the region of interest are usually regarded as a measure of the FC when calculating FCs between different brain regions (Koelsch et al., 2018; Meichen et al., 2018; Rubia et al., 2019). This is mainly because the inherent noise in fMRI data is challenging to characterize the properties of individual voxels, and the current methods cannot flexibly analyze the FC differences of voxel levels. To overcome this problem, Baldassano et al. (2012) proposed a new FC method that incorporates a spatial smoothness constraint using regularized optimization, enabling the discovery of voxel-level interactions between brain regions from the small datasets characteristic of fMRI experiments. But not all voxels in the brain regions are involved in the corresponding neural activity. Recently, Armañanzas et al. (2017) directly used the activated voxels of the brain in different machine learning techniques to tackle the automatic pattern analysis of Alzheimer disease patients and healthy individuals, which obtained a high classification accuracy.

In this paper, the activated FCs between different brain regions are used to reveal the changes of brain functional activity caused by professional particularity of seafarers. There are three main steps: firstly, the activated voxels in the fMRI data are extracted using the spatial ICA method and the corresponding activated brain regions are determined according to the Anatomical Automatic Labeling (AAL) template. Then, the FCs between the activated voxels are constructed using Pearson correlation. Finally, the accurate classification of seafarers and non-seafarers is achieved through SVM using the FCs between common activated voxels of seafarer and non-seafarer groups as well as the FCs with significant differences between them. The results show that the regularity of neural activity in seafarer’s brain has its own specificity compared with those in non-seafarers, which has a great significance for the study of brain plasticity and brain health of seafarers.

## Materials and Methods

### Data Acquisition

The resting-state fMRI data of 20 male professional seafarers (ages 42–57 years, mean age 49 years old, right-handed) recruited from a shipping company of Shanghai were involved in this study. They came from various positions, such as mate, helmsman, and seaman, and all of them had about 10–20 years of experience in navigation. Meanwhile, 20 Chinese male participants (ages 48–55 years, mean age 51 years old, right-handed) recruited from land-based jobs in the university or secondary school campus were used as the non-seafarer control group. All the subjects in the non-seafarer group were without navigational skills, maritime professional training, or long-term experience at sea. The ages of the subjects in the two groups were matched, and the education levels between them were equivalent. The studies involving human participants were reviewed and approved by the Independent Ethics Committee of East China Normal University. The participants provided written informed consent to participate in this study. The participants had no reported history of neurological or psychiatric disorders. In the process of fMRI data acquisition, all the participants were instructed to keep the body motionless, eyes closed, relaxed without thinking anything, and awake, and their ears were stuffed up with earplugs in order to reduce the effect of the machine noise. The fMRI data collection task was completed in the Shanghai Key Laboratory of Magnetic Resonance of the East China Normal University, which was acquired using a gradient echo planar imaging (EPI) with 36 slices providing whole-brain coverage and 160 volumes, a repetition time of 2.0 s and a scan resolution of 64 × 64, the in-plane resolution was 3.75 mm × 3.75 mm, and the slice thickness was 4 mm. This dataset was also reported in our previous research (Wang et al., 2017, 2018).

### Data Preprocessing

In the experiment, the fMRI data were preprocessed by using the DPARSF software^1^, including removing the first 10 time points in the fMRI data of each subject for magnetization equilibrium to compensate for transient scanner instability and participant’s adaptation to the circumstance, slice timing, motion correction, spatial normalization, and spatial smoothing with the Gaussian kernel set to 4 mm, band-pass filtering (0.01–0.08 Hz), and detrending. Meanwhile, the head motion, whole-brain signal, cerebrospinal fluid, and white matter signals were removed as nuisance covariates by a multiple linear regression analysis to reduce the effect of the physiological artifacts, motion, and non-neuronal blood oxygen level-dependent fluctuations. Particularly, spatial ICA was implemented using FastICA algorithm (Hyvarinen and Oja, 1997) in all experiments. Moreover, ICASSO (Himberg et al., 2004) with 20 runs of ICA was used to obtain reliable independent components (ICs), and minimum description length (MDL) (Li et al., 2007) was used to estimate the number of components. Furthermore, the location and display of these networks were assessed by using the MRIcro software^2^.

### Methods

In the following, the resting-state fMRI data of 20 seafarers and 20 age-matched healthy subjects were denoted as the seafarer and non-seafarer groups, respectively, and the same three steps were implemented on the group of seafarer and non-seafarer separately. Firstly, ICA was performed on each individual of each group, and the corresponding activated voxels of each subject were obtained through the z-threshold method at a given threshold value so as to obtain the common activated voxels corresponding to all subjects in each group. Then, AAL template was used to locate the brain regions in the cerebral cortex corresponding to the common activated voxels of each group, and the brain regions in which the number of voxels was greater than 10 and exceeded 1% of the total voxels in the region were selected for analysis.

Next, the time series of activated voxels were used to calculate the FCs of each subject between the common activated voxels corresponding to the subjects of each group, and the statistical analysis of each FC between these two groups was conducted by the two-sample *T*-test with false discovery rate (FDR) correction to find out the FCs with significant differences. Subsequently, the distribution of the activated voxels corresponding to these FCs with significant differences in the AAL brain region was statistically analyzed. Finally, all FCs between the common activated voxels and the FCs with significant differences were used as the features of SVM for the classification of seafarers and non-seafarers, which verified the ability of FCs with significant differences for seafarers’ identification.

## Results and Analysis

**FIGURE 1:**
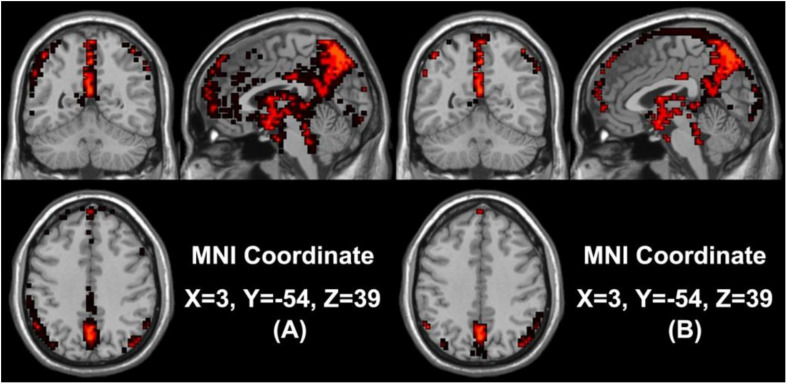
**(A)** The spatial brain maps of activated voxels in the non-seafarer group. **(B)** The spatial brain map of activated voxels in the seafarer group. The red represents the common activated regions of the non-seafarer and seafarer groups, and the black represents the individually activated regions of each group.

In our study, we mainly considered the activated voxels in the gray matter region of the brain. Figure 1 presents the spatial brain maps of corresponding activated voxels in non-seafarer and seafarer groups, as shown in Figures 1A,B, respectively. Among them, the red represents the common activated regions of non-seafarer and seafarer groups, and the black represents the individual activated regions of each group. The map is obtained with a threshold | z| ≥ 2 after z-scoring the ICs of spatial ICA. To accurately quantify the distribution of these activating voxels in the brain, we show the statistical distribution of activated voxels of non-seafarer and seafarer groups in the AAL brain regions of the cerebral cortex and their corresponding Montreal Neurological Institute (MNI) coordinates in Table 1. Each brain region contains more than 10 activated voxels that account for more than 1% of the total voxels in the region.

**TABLE 1:** The statistical distribution of activated voxels of the non-seafarer group and seafarer group in the Anatomical Automatic Labeling (AAL) brain regions and their corresponding Montreal Neurological Institute (MNI) coordinates.

AAL regions	MNI coordinates	Non-seafarer group		Seafarer group	
			
		Number	Ratio	Number	Ratio
Frontal_Sup_L	(−18.45, 34.81, 42.2)	11	0.0102		
Frontal_Sup_R	(21.9, 31.12, 43.82)	14	0.0121		
Frontal_Mid_R	(37.59, 33.06, 34.04)	10	0.0066		
Frontal_Inf_Tri_L	(−45.58, 29.91, 13.99)	20	0.0275		
Supp_Motor_Area_L	(−5.32, 4.85, 61.38)			16	0.0244
Supp_Motor_Area_R	(8.62, 0.17, 61.85)			26	0.0390
Frontal_Sup_Medial_L	(−4.8, 49.17, 30.89)	69	0.0816	24	0.0284
Frontal_Sup_Medial_R	(9.1, 50.84, 30.22)	46	0.0718	26	0.0406
Cingulum_Ant_L	(−4.04, 35.4, 13.95)	33	0.0775		
Cingulum_Mid_L	(−5.48, −14.92, 41.57)	17	0.0275		
Cingulum_Mid_R	(8.02, −8.83, 39.79)	19	0.0314		
Cingulum_Post_L	(−4.85, −42.92, 24.67)	21	0.1533		
Cingulum_Post_R	(7.44, −41.81, 21.87)	11	0.1264		
ParaHippocampal_L	(−7.63, −25.36, 70.07)	12	0.0420		
ParaHippocampal_R	(25.38, −15.15, −20.47)	25	0.0791		
Calcarine_L	(−7.14, −78.67, 6.44)	25	0.0386	17	0.0262
Calcarine_R	(15.99, −73.15, 9.4)	14	0.0258		
Cuneus_L	(−5.93, −80.13, 27.22)	45	0.1002	14	0.0312
Lingual_R	(16.29, −66.93, −3.87)	38	0.0560		
Occipital_Mid_L	(−32.39, −80.73, 16.11)	11	0.0115		
Occipital_Mid_R	(37.39, −79.7, 19.42)	13	0.0218		
Parietal_Sup_L	(−23.45, −59.56, 58.96)	56	0.0887	11	0.0174
Parietal_Sup_R	(26.11, −59.18, 62.06)	44	0.0680	28	0.0433
Parietal_Inf_L	(−42.8, −45.82, 46.74)	49	0.0704	39	0.0560
Parietal_Inf_R	(46.46, −46.29, 49.54)	95	0.2267		
SupraMarginal_R	(57.61, −31.5, 34.48)	49	0.0891		
Angular_L	(−44.14, −60.82, 35.59)	33	0.0965	34	0.0994
Angular_R	(45.51, −59.98, 38.63)	104	0.2097	38	0.0766
Precuneus_L	(−42.46, −22.63, 48.92)	238	0.2206	154	0.1427
Precuneus_R	(9.98, −56.05, 43.77)	169	0.1807	206	0.2203
Paracentral_Lobule_L	(−7.63, −25.36, 70.07)			19	0.0450
Paracentral_Lobule_R	(7.48, −31.59, 68.09)			32	0.1410
Caudate_L	(−11.46, 11, 9.24)	19	0.0683		
Caudate_R	(14.84, 12.07, 9.42)	21	0.0739		
Thalamus_R	(13, −17.55, 8.09)	10	0.0326		
Temporal_Sup_L	(−53.16, −20.68, 7.13)	11	0.0164		
Temporal_Sup_R	(58.15, −21.78, 6.8)	24	0.0249		
Temporal_Pole_Sup_L	(−39.88, 15.14, −20.18)	41	0.1073		
Temporal_Pole_Sup_R	(48.25, 14.75, −16.86)	35	0.0875	21	0.0525
Temporal_Mid_R	(57.47, −37.23, −1.47)	22	0.0162		

It can be seen from the table that there are no voxels activated in the brain regions of Frontal_Sup (L/R), Frontal_Mid_R, Frontal_Inf_Tri_L, Cingulum_Ant_L, Cingulum_Mid (L/R), Cingulum_Post (L/R), ParaHippocampal (L/R), Calcarine_R, Lingual_R, Occipital_Mid (L/R), Parietal_Inf_R, SupraMarginal_R, Caudate (L/R), Thalamus_R, Temporal_Sup (L/R), Temporal_Pole_Sup_R, and Temporal_Mid_R in the seafarer group compared with those of the non-seafarer group. In contrast, several brain areas are individually activated in the seafarer group, such as Supp_Motor_Area (L/R) and Paracentral_Lobule (L/R). In addition, the number of common activated voxels of the non-seafarer and seafarer groups is 544, and they are mainly located in the brain regions of Frontal_Sup_Medial (L/R), Calcarine_L, Cuneus_L, Parietal_Sup (L/R), Parietal_Inf_L, Angular (L/R), Precuneus (L/R), and Temporal_Pole_Sup_R. Moreover, it can be found that the number of activated voxels in the non-seafarer group is more than that in the seafarer group in most of these brain regions.

**FIGURE 2:**
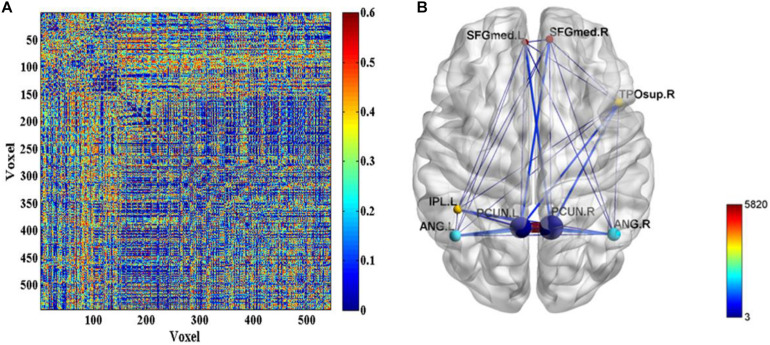
**(A)** The *p*-values of *t*-test on the functional connectivities (FCs) between the common activated voxels of the seafarer and non-seafarer groups. **(B)** The spatial brain network between Anatomical Automatic Labeling (AAL) regions with activated voxels of FCs with significant differences.

Further, the FC differences between these common activated voxels were compared between non-seafarer and seafarer groups. Figure 2A shows statistical test results on the FCs between the common activated voxels of the seafarer and non-seafarer groups, which are obtained using *t*-test with a confidence level of 95% after FDR correction. Figure 2B shows the spatial brain network between AAL regions corresponding to the activated voxels of FCs with significant differences, where the size of the edges between different AAL regions represents the number of FCs with significant differences between common activated voxels. Particularly, the voxel number and ratio of these AAL regions and their corresponding MNI coordinates are shown in Table 2, which are more than 10 voxels and account for more than 1% of the total voxels in the region.

**TABLE 2:** The statistical distribution of activated voxels corresponding to functional connectivities (FCs) with significant differences between the seafarer and non-seafarer groups in the Anatomical Automatic Labeling (AAL) brain regions as well as the Montreal Neurological Institute (MNI) coordinates.

AAL regions	MNI coordinates	Voxel number	Voxel ratio
Frontal_Sup_Medial_L	(−4.8, 49.17, 30.89)	10	0.0118
Frontal_Sup_Medial_R	(9.1, 50.84, 30.22)	11	0.0172
Parietal_Inf_L	(−42.8, −45.82, 46.74)	17	0.0244
Angular_L	(−44.14, −60.82, 35.59)	15	0.0439
Angular_R	(45.51, −59.98, 38.63)	27	0.0544
Precuneus_L	(−42.46, −22.63, 48.92)	119	0.1103
Precuneus_R	(9.98, −56.05, 43.77)	122	0.1305
Temporal_Pole_Sup_R	(48.25, 14.75, −16.86)	13	0.0325

**FIGURE 3:**
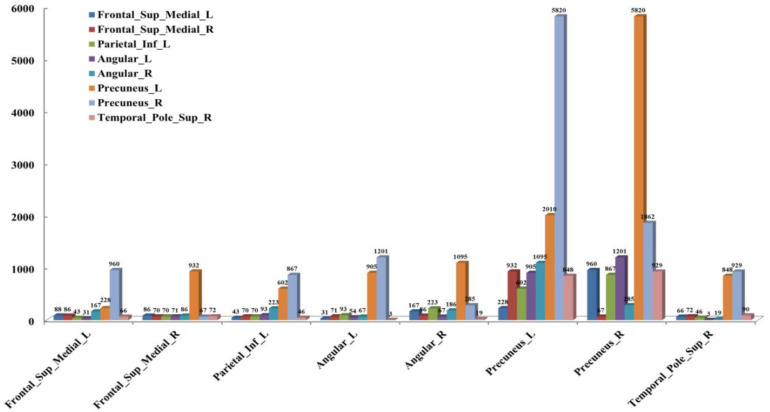
The number of functional connectivities (FCs) between the Anatomical Automatic Labeling (AAL) regions corresponding to the activated voxels of FCs with significant differences.

According to the analysis results of the above statistical comparison, we can calculate the number of FCs with significant differences among the common activated voxels between different AAL regions, which is shown in Figure 3. It can be seen clearly from the figure that the number of FCs between Precuneus_R with Frontal_Sup_Medial_R, Parietal_Inf_L, Angular_L, Angular_R, Precuneus_L, Precuneus_R and Temporal_Pole_Sup_R, Precuneus_L with Frontal_Sup_Medial_L, Parietal_Inf_L, Angular_L, Precuneus_L, Precuneus_R, and Temporal_Pole_Sup_R is higher than that between other regions, while there is almost no FC between Angular_L and Temporal_Pole_Sup_R. Moreover, it can be further found from the figure that there are many FCs with significant differences between common activated voxels of the non-seafarer and seafarer groups in the AAL brain regions of Precuneus_L and Precuneus_R, which means that the function of the Precuneus may be altered by occupation.

**FIGURE 4:**
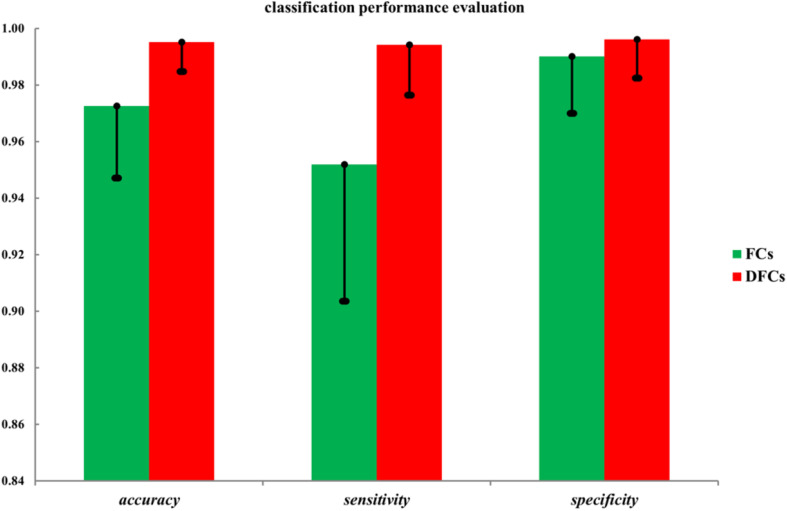
The classification accuracy, sensitivity, and specificity using all functional connectivities (FCs) and FCs with significant differences (DFCs) between the common activated voxels of the seafarer and non-seafarer groups.

Finally, to evaluate the role of these FCs in the seafarer identification process, we use all FCs and FCs with significant differences between the common activated voxels as the features of SVM for the classification of seafarers and non-seafarers, and the classification accuracy, sensitivity, and specificity are shown in Figure 4. In particular, the value of parameter C = 1 and linear kernel function were used in the SVM, and the 10-fold cross-validation was used to control the overfitting problem. SVM was run 100 times with a test ratio of 0.2 when using it for classification. It can be seen clearly from the figure that the classification accuracy, sensitivity, and specificity obtained using FCs with significant differences are significantly higher than those obtained using all FCs between the common activated voxels of the seafarer and non-seafarer groups, which means that the FCs with significant differences between the common activated voxels play an important role in the classification of seafarers and non-seafarers and has a potential significance for the seafarer neural pathogenesis research.

## Conclusion and Discussion

In view of the current situation that the fMRI-based brain FC analysis mainly focuses on the large-scale brain regions, this study investigated the brain FC between the activated voxels of seafarers and non-seafarers. First, the distribution of activated voxels of seafarers and non-seafarers obtained by ICA method in the AAL brain region was analyzed in depth. Subsequently, the two-sample *T*-test was used to statistically analyze the FCs between the commonly activated voxels of the two groups, and the FCs with significant differences were obtained. Finally, using these FCs as classified features, SVM verified that FCs with significant differences between the commonly activated voxels had better recognition performance on seafarers and non-seafarers, which demonstrated that FCs between activated voxels can better reveal the differences of neurological activities between seafarers and non-seafarers.

According to the distribution of activated voxels of seafarer and non-seafarer groups in the AAL brain regions as shown in Table 1, the results showed that many brain regions of the superior frontal gyrus, middle frontal gyrus, inferior frontal gyrus, anterior cingulate and paracingulate gyri, median cingulate and paracingulate gyri, posterior cingulate gyrus, parahippocampal gyrus, calcarine fissure and surrounding cortex, lingual gyrus, middle occipital gyrus, inferior parietal, supramarginal gyrus, caudate nucleus, thalamus, superior temporal gyrus, middle temporal gyrus, and superior temporal gyrus (temporal pole) were not activated in the seafarer group compared with the non-seafarer group, except for the brain regions of supp_motor_area (L/R) and paracentral_lobule (L/R), which were only activated in the seafarer group. They were mainly located in the prefrontal lobe, frontal lobe, parietal lobe, temporal lobe, occipital lobe, and subcortical lobe, and there are more voxels in most of the brain regions of the non-seafarer group.

Among them, the supplementary motor area is located on the midline surface of the hemisphere just in front of the primary motor cortex that contributes to the control of movement including the control of postural stability during stance or walking and bimanual coordination (Serrien et al., 2002). The paracentral lobule is located on the medial surface of the hemisphere and the anterior portion of the paracentral lobule is often referred to as the supplementary motor area. When seafarers are in the process of sea navigation, they are in a state of turbulence for a long time due to the influence of marine environmental conditions and its own factors. Therefore, it requires extra effort to maintain body balance (such as swinging hands) when walking on the ship, which results in more active neural activities in the relevant brain regions. This may be why these two brain regions are activated separately in the seafarer’s brain.

The frontal lobe is located at the front of each hemisphere, which is associated with reward, attention, short-term memory tasks, planning, and motivation (Lu et al., 2004). As a part of the frontal lobe, the prefrontal lobe has been implicated in planning complex cognitive behavior, personality expression, decision-making, and moderating social behavior (Yang and Raine, 2009). The temporal lobe is involved in processing sensory input into derived meanings for the appropriate retention of visual memory, language comprehension, and emotion association (Hickok and Poeppel, 2007). The occipital lobe is the visual processing center of the mammalian brain that contains a low-level description of the local orientation, spatial frequency, and color properties within small receptive fields (Chilosi et al., 2006). Compared with non-seafarers, seafarers’ living and working conditions at sea are quite different from those on land, such as narrow living space, monotonous work content, and the loneliness of being away from their families for a long time. These factors limited their neural activity of cognitive functions in the associated brain regions and thus showed significantly weaker activation than that of non-seafarers, resulting in the inactivation of these brain regions in the seafarer group.

Moreover, the results in Figure 4 showed that the identification performance using only FCs with significant differences between the activated voxels was better than that using all FCs between the activated voxels in the classification of seafarers and non-seafarers. According to the distribution of the activated voxels related to FCs with significant differences shown in Table 2, they were mainly located in the brain regions of the superior frontal gyrus, inferior parietal gyrus, angular gyrus, precuneus, and superior temporal gyrus. Based on the statistics of the number of FCs with significant differences between different brain regions in Figure 3, we found that the FCs between activated voxels were mainly located between the Precuneus_L and Precuneus_R as well as between them with other brain regions. Especially for Precuneus_L and Precuneus_R, which may mean that they played an important role in the classification prediction of seafarers, and they were consistent with the results in Figure 2.

The precuneus is part of the superior parietal lobule on the medial surface of the cerebral hemisphere. It is involved with episodic memory, visuospatial processing, reflections upon self, and aspects of consciousness. In addition, it also has been considered as the “core node” or “hub” of the default mode network that is activated during “resting consciousness” in which people will not consciously participate in sensory or motor activities (Cavanna and Andrea, 2007). While the survival environment faced by seafarers at sea is very likely to affect the functional activity of these brain regions (Wu et al., 2020). For example, the monotonous work content weakens the seafarers’ memory-related brain functional activities and the adverse weather conditions require that seafarers pay more attention to the atmosphere’s space–time changes. The boring living conditions and the troubles of staying away from their families for a long time are easily to have an impact on seafarers’ psychology, which is reflected on the abnormal changes of seafarers’ brain functional activities in terms of self-consciousness, so that the FCs of these brain regions show significant differences between seafarers and non-seafarers. Therefore, it is worth to further study in the future, which may be providing a new perspective for the study of seafarers’ brain neural activity.

## Data Availability Statement

The raw data supporting the conclusions of this article will be made available by the authors, without undue reservation.

## Ethics Statement

The studies involving human participants were reviewed and approved by Independent Ethics Committee of East China Normal University. The patients/participants provided their written informed consent to participate in this study.

## Author Contributions

YS, WZ, and NW contributed to the acquisition of fMRI data. YS contributed to the design of the work and drafting of the article. YS and NW contributed to the analysis and interpretation. All authors contributed to the article and approved the submitted version.

## Conflict of Interest

The authors declare that the research was conducted in the absence of any commercial or financial relationships that could be construed as a potential conflict of interest.

## Footnotes

1 http://rfmri.org/DPARSF

2 http://www.mricro.com
